# Effects of Loading and Boundary Conditions on the Performance of Ultrasound Compressional Viscoelastography: A Computational Simulation Study to Guide Experimental Design

**DOI:** 10.3390/ma14102590

**Published:** 2021-05-16

**Authors:** Che-Yu Lin, Ke-Vin Chang

**Affiliations:** 1 Institute of Applied Mechanics, College of Engineering, National Taiwan University, No. 1, Sec. 4, Roosevelt Road, Taipei 10617, Taiwan; 2 Department of Physical Medicine and Rehabilitation, National Taiwan University Hospital and National Taiwan University College of Medicine, Taipei 100, Taiwan; kvchang011@gmail.com; 3 Department of Physical Medicine and Rehabilitation, National Taiwan University Hospital, Bei-Hu Branch, Taipei 10845, Taiwan

**Keywords:** elastography, mechanical properties, viscoelastic properties, creep, stress relaxation

## Abstract

Most biomaterials and tissues are viscoelastic; thus, evaluating viscoelastic properties is important for numerous biomedical applications. Compressional viscoelastography is an ultrasound imaging technique used for measuring the viscoelastic properties of biomaterials and tissues. It analyzes the creep behavior of a material under an external mechanical compression. The aim of this study is to use finite element analysis to investigate how loading conditions (the distribution of the applied compressional pressure on the surface of the sample) and boundary conditions (the fixation method used to stabilize the sample) can affect the measurement accuracy of compressional viscoelastography. The results show that loading and boundary conditions in computational simulations of compressional viscoelastography can severely affect the measurement accuracy of the viscoelastic properties of materials. The measurement can only be accurate if the compressional pressure is exerted on the entire top surface of the sample, as well as if the bottom of the sample is fixed only along the vertical direction. These findings imply that, in an experimental validation study, the phantom design should take into account that the surface area of the pressure plate must be equal to or larger than that of the top surface of the sample, and the sample should be placed directly on the testing platform without any fixation (such as a sample container). The findings indicate that when applying compressional viscoelastography to real tissues in vivo, consideration should be given to the representative loading and boundary conditions. The findings of the present simulation study will provide a reference for experimental phantom designs regarding loading and boundary conditions, as well as guidance towards validating the experimental results of compressional viscoelastography.

## 1. Introduction

Ultrasound elastography is an ultrasound-based imaging method for noninvasively measuring parameters related to the stiffness of materials [1,2,3]. This imaging technology was first described in the early 1990s [4] and subsequently developed into a real-time method for obtaining the map of parameters related to the stiffness of materials [1]. Ultrasound elastography is a clinical technique used to diagnose pathological conditions of various tissues [5], such as breast [6], liver [7], prostate [8], thyroid [9], tendons [10], muscles [11], and heel pads [12,13,14]. In addition, ultrasound elastography can potentially be used to evaluate the properties of a biomaterial for monitoring its development to ensure its quality [15,16,17]. It is based on the fact that pathological processes often cause changes in the stiffness of tissues [18,19], and the properties of a biomaterial are often related to its stiffness [15,20]. However, most tissues and biomaterials are viscoelastic [15]. This means that they have both viscous (fluid) and elastic (solid) properties [21]. Changes in the status of tissues and biomaterials often lead to alterations in both the fluid and solid properties. Therefore, it is important to characterize the viscoelastic properties if we intend to completely evaluate pathological tissues or the condition of an engineered biomaterial. In many circumstances, measuring the stiffness alone may not be sufficient to completely evaluate the status of tissues and biomaterials. Several studies have shown that viscosity is a better discriminator than stiffness to differentiate between malignant and benign tumors [22,23]. One study also suggested that considering viscosity can provide additional important information, rather than just considering stiffness alone [24].

**Figure 1:**
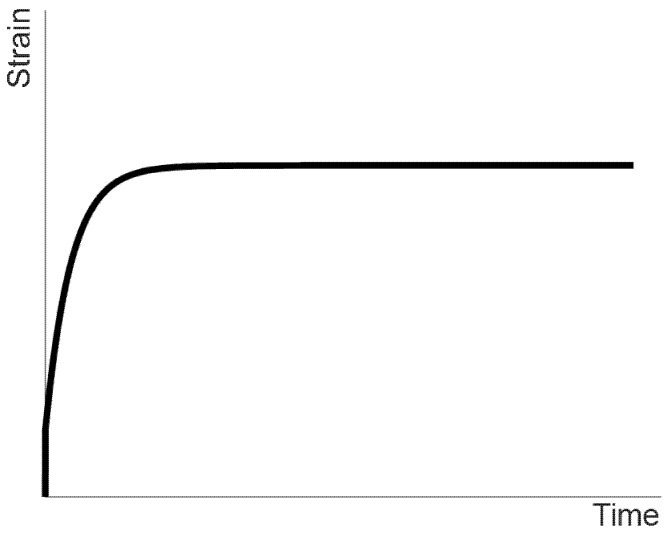
Illustration of creep behavior (increasing strain over time).

In a laboratory setting, the viscoelastic properties of materials are often evaluated by mechanical material testing systems. Several ultrasound techniques have been designated by different research groups to evaluate the viscoelastic properties of materials based on analyzing the creep behavior (increasing strain over time, Figure 1), generally called viscoelastic creep imaging [25]. In viscoelastic creep imaging, the creep behavior of an element inside the material can be acquired by applying a constant stress to the material. The constant stress can be induced by ultrasound acoustic radiation force [16,17,26,27,28,29,30,31,32], or by external mechanical compression on the top surface of the material [33,34]. If a viscoelastic mathematical model is used to curve-fit the creep behavior, the viscoelastic properties of materials can be quantitatively evaluated. In the design and experimental setup of viscoelastic creep imaging, several factors should be carefully considered to achieve the best measurement accuracy and optimal performance of the system.

The present study will investigate the measurement accuracy of compressional viscoelastography (a type of viscoelastic creep imaging using external mechanical compression as the source of excitation) through computational simulations of imaging. The aim of the present study is to use finite element analysis to investigate the performance of compressional viscoelastography to measure the viscoelastic properties of homogeneous viscoelastic materials, and to investigate how loading conditions (the distribution of the applied compressional pressure on the surface of the sample) and boundary conditions (the fixation method used to stabilize the sample) can affect the measurement accuracy. The results of the present simulation study provide a reference for experimental phantom designs regarding loading and boundary conditions, as well as guidance towards validating the experimental results of compressional viscoelastography.

## 2. Materials and Methods

### 2.1. Principle of Compressional Viscoelastography

**Figure 2:**
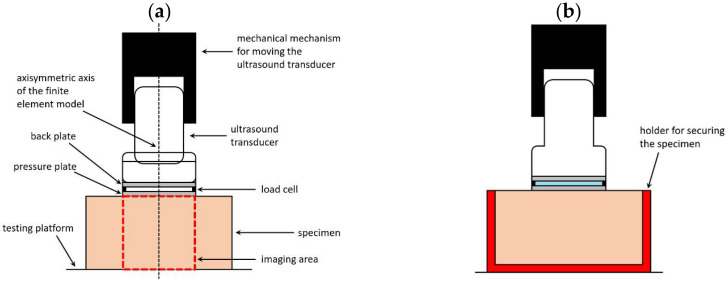

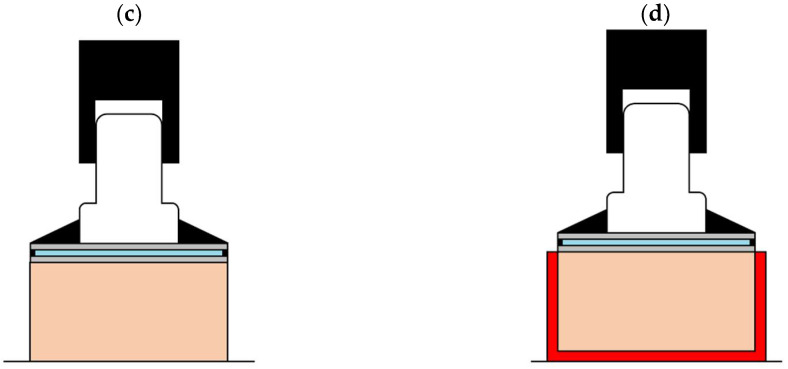
The design of a compressional viscoelastography system with different experimental settings. (**a**) The surface area of the pressure plate is half of that of the top surface of the sample, and the sample is placed directly on the testing platform without any fixation. This setup is associated with the condition in Figure 5d. (**b**) The surface area of the pressure plate is half of that of the top surface of the sample, and the sample is secured by a sample container. This setup is associated with the condition in Figure 5f. (**c**) The surface area of the pressure plate is equal to that of the top surface of the sample, and the sample is placed directly on the testing platform without any fixation. This setup is associated with the condition in Figure 5a. (**d**) The surface area of the pressure plate is equal to that of the top surface of the sample, and the sample is secured by a sample container. This setup is associated with the condition in Figure 5c.

The design of the compressional viscoelastography system is shown in Figure 2 (refer to references [33,34] for a more detailed introduction of the design of this system). The system uses a circular pressure plate to apply a uniform compressional pressure on the surface of the material. The pressure and back plates are connected to each other and attached to the ultrasound transducer. The magnitude of the compressional pressure can be measured by load cells attached between the two plates. The system has a feedback control unit that allows for the simultaneous monitoring and control of the compressional pressure. The movement of the transducer is controlled by a stepper motor. The strain of an element within the material is measured using ultrasound imaging.

**Figure 3:**
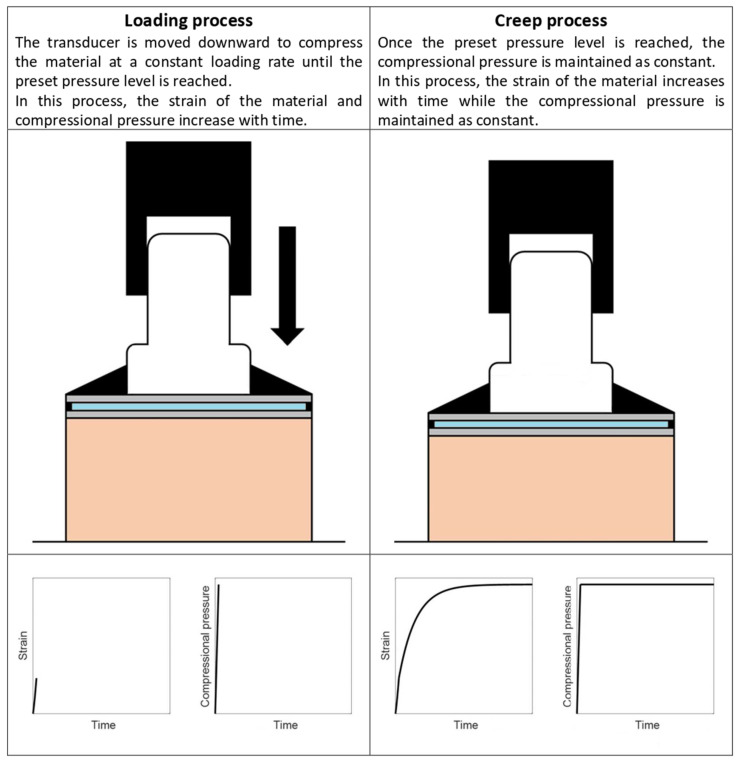
Illustration of the measurement processes of compressional viscoelastography.

In the measurement, the transducer is moved downward to compress the material at a constant loading rate until the preset pressure level is reached. The loading rate must be high to mimic a step load. Once the preset pressure level is reached, the compressional pressure is maintained as constant through a feedback control system. During the period when the compressional pressure is maintained as constant, the stress of an element within the material is constant as well. Therefore, each element within the material exhibits creep behavior. The measurement processes are illustrated in Figure 3.

**Figure 4:**
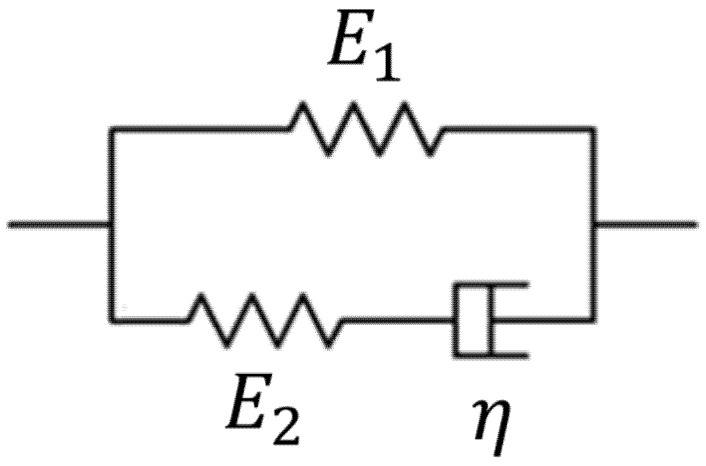
Illustration of the Maxwell representation of the standard linear solid model. $E_{1}$, $E_{2}$, and η are three viscoelastic properties.

In the present study, the Maxwell representation of the standard linear solid model (hereafter referred to as the standard linear solid model for brevity), as shown in Figure 4, is used to describe the viscoelastic behaviors of materials. If a step stress excitation is used to excite the material, the creep behavior (increasing strain over time) described by the standard linear solid model is [35]:

(1) $$\epsilon \left(t\right)=\frac{\sigma_{0}}{E_{1}}\left(1-\frac{E_{2}}{E_{1}+E_{2}}\cdot e^{-\frac{1}{\tau_{c}}t}\right)$$

where ϵ(t) is the strain over time of an element within the material; $\tau_{C}=\eta \left(E_{1}+E_{2}\right)/E_{1}E_{2}$ is the creep time constant (also called the retardation time constant); $E_{1}$, $E_{2}$, and η are parameters relevant to viscoelastic properties; $\sigma_{0}$ is the stress value of an element within the material following the step stress excitation at t=0 (i.e., the beginning of creep); and $\sigma_{0}$ is the constant stress value during creep. In the present study, $\sigma_{0}$ in Equation (1) is assumed to be the magnitude of uniform compressional pressure applied on the surface of the material, although this assumption may not be perfectly accurate for each element within the material since the appropriateness of this assumption depends on the loading and boundary conditions.

If Equation (1) is used to curve-fit the creep curve of each element within the material, the $E_{1}$, $E_{2}$, and $\tau_{C}$ of each element can be quantitatively evaluated. Consequently, the spatial distribution of the viscoelastic properties of the material can be obtained, as described in the following section.

### 2.2. Computational Simulations and Data Analysis

Finite element analysis using ABAQUS 2019 (Dassault Systems, Simulia Corporation, Johnson, RI, USA) is used to investigate the measurement accuracy of compressional viscoelastography when measuring the viscoelastic properties of the material under different loading and boundary conditions.

**Figure 5:**
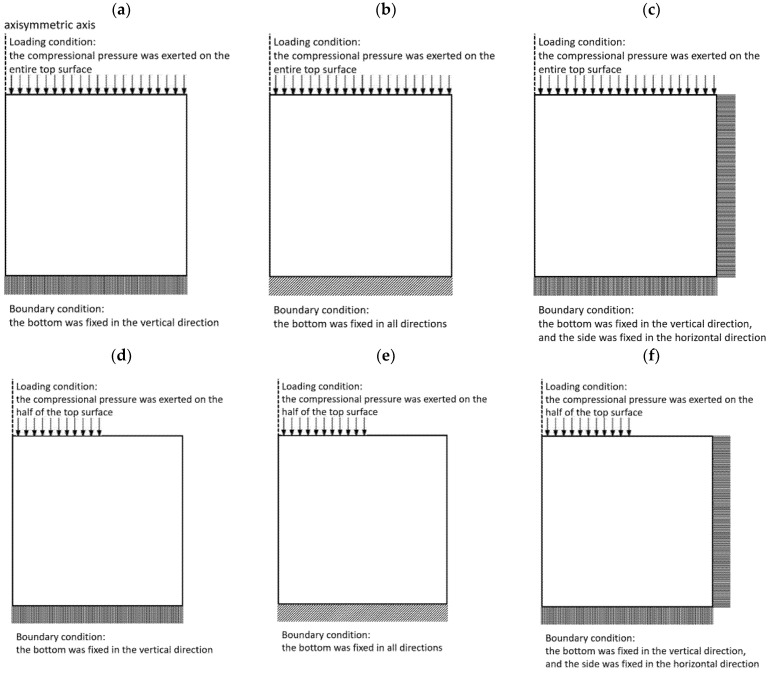
Illustration of the loading and boundary conditions in each simulation test. (**a**) The uniform compressional pressure is exerted on the entire top surface. The bottom is fixed along the vertical direction while the side is not fixed. (**b**) The uniform compressional pressure is exerted on the entire top surface. The bottom is fixed along all directions while the side is not fixed. (**c**) The uniform compressional pressure is exerted on the entire top surface. The bottom is fixed along the vertical direction while the side is fixed along the horizontal direction. (**d**) The uniform compressional pressure is exerted on half of the top surface. The bottom is fixed along the vertical direction while the side is not fixed. (**e**) The uniform compressional pressure is exerted on half of the top surface. The bottom is fixed along all directions while the side is not fixed. (**f**) The uniform compressional pressure is exerted on half of the top surface. The bottom is fixed along the vertical direction while the side is fixed along the horizontal direction.

Six simulation tests were run in this study. The specific settings regarding the loading and boundary conditions in each simulation test are described below and illustrated in Figure 5:

(1) Simulation test 1 (Figure 5a): uniform compressional pressure is exerted on the entire top surface. The bottom is fixed along the vertical direction while the side is not fixed. This condition is associated with the experimental setup in Figure 2c. Simulation test 1 is also used to investigate the validity of compressional viscoelastography. See Appendix A for the result of the validity test.
(2) Simulation test 2 (Figure 5b): uniform compressional pressure is exerted on the entire top surface. The bottom is fixed along all directions while the side is not fixed.
(3) Simulation test 3 (Figure 5c): uniform compressional pressure is exerted on the entire top surface. The bottom is fixed along the vertical direction while the side is fixed along the horizontal direction. This condition is associated with the experimental setup in Figure 2d.
(4) Simulation test 4 (Figure 5d): uniform compressional pressure is exerted on half of the top surface. The bottom is fixed along the vertical direction while the side is not fixed. This condition is associated with the experimental setup in Figure 2a.
(5) Simulation test 5 (Figure 5e): uniform compressional pressure is exerted on half of the top surface. The bottom is fixed along all directions while the side is not fixed.
(6) Simulation test 6 (Figure 5f): uniform compressional pressure is exerted on half of the top surface. The bottom is fixed along the vertical direction while the side is fixed along the horizontal direction. This condition is associated with the experimental setup in Figure 2b.

The six simulation tests are different in their loading and boundary conditions as described above, but they have some common settings. In each simulation test, an axisymmetric finite element model is used, and the radius and thickness of the axisymmetric model are 50 mm and 50 mm, respectively. Square finite elements (0.5 mm × 0.5 mm) are used to mesh the model. The radius and thickness of the imaging region (the region of the model where data are taken to produce the image, which is chosen as the region of the model just below the transducer, with a length of 50 mm) are 25 mm and 50 mm, respectively, as shown in Figure 2a.

The model is made of linear viscoelastic material. The material is also assumed to be incompressible, isotropic, and homogeneous. The mechanical properties of the material are defined by four properties, including the modulus of elasticity (E), Poisson’s ratio (set as a constant of 0.495, the maximum Poisson’s ratio that can be set in ABAQUS), and two parameters in the one-branch dimensionless relaxation modulus:

(2) $$g_{R}\left(t\right)=1-g\left(1-e^{\frac{-t}{\tau_{R}}}\right)$$

where g is a material constant (0<g<1), and $\tau_{R}$ is the relaxation time constant.

The loading rate for applying the compressional pressure is designed to be high (the time duration from zero to maximum pressure is 1/6 s) to mimic a step load. Once the maximum pressure (set as a constant of 1000 Pa in each simulation test) is reached, the maximum pressure is then maintained as a constant for a period of time. During this period, each element in the model exhibits creep behavior; that is, the strain response of each element increases with time until the steady state is reached. The creep curve of each element is sent to MATLAB (R2019a, Mathworks, Natick, MA, USA) for analysis. The $E_{1}$, $E_{2}$, and $\tau_{C}$ of each element can be obtained by using Equation (1) to curve-fit the creep curve of each element. η can then be obtained by using the definition of the creep time constant $\tau_{C}=\eta \left(E_{1}+E_{2}\right)/E_{1}E_{2}$. It has been reported that E is equal to $E_{1}$, while 𝑔 is equal to $E_{2}/\left(E_{1}+E_{2}\right)$, and $\tau_{R}$ is equal to $\eta /E_{2}$ [35]. Consequently, the three mechanical properties of each element (E, g, and $\tau_{R}$) set in ABAQUS can be obtained.

Once the value of a mechanical property (E, g, or $\tau_{R}$) of each element is assigned with a specific color, the 2D spatial distribution map of that mechanical property of the axisymmetric image region can be constructed (since the image region is quadrilateral and the elements within are square). However, this map of the axisymmetric image region is just half of the map that should be obtained in reality (since the model is axisymmetric). Therefore, this map of the axisymmetric image region (called the original map here) is reflected, and then combined with the original map to produce a full map.

The corresponding error map is also constructed for each mechanical property. The error value at each element in the error map is calculated by:

(3) $$\mathrm{error}=\frac{\left|\mathrm{simulation}\;\mathrm{value}-\mathrm{theoretical}\;\mathrm{value}\right|}{\mathrm{theoretical}\;\mathrm{value}}$$

where the simulation value is the value of the mechanical property of an element obtained from the simulation, and the theoretical value is the value of the mechanical property set in ABAQUS. If the error is less than 10% [36], the measurement is considered to be accurate.

In each simulation test, the mechanical properties are fixed and set as E = 10 kPa, $\tau_{R}$ = 5 s, and g = 0.8 (because, in our preliminary study, it was found that the pattern of the map of a mechanical property is similar regardless of the mechanical properties; in other words, the pattern of the map of a mechanical property is sensitive to the loading and boundary conditions but insensitive to the mechanical properties). In the 2D spatial distribution map of each mechanical property in each simulation test, the percentage of the region in the map consisting of elements having the simulation value within ±10% [36] of the theoretical value set in ABAQUS is calculated. The larger this percentage, the more accurate the measurement, because the wider the region in the 2D spatial distribution map, the more accurate the value of the mechanical property.

## 3. Results

**Figure 6:**
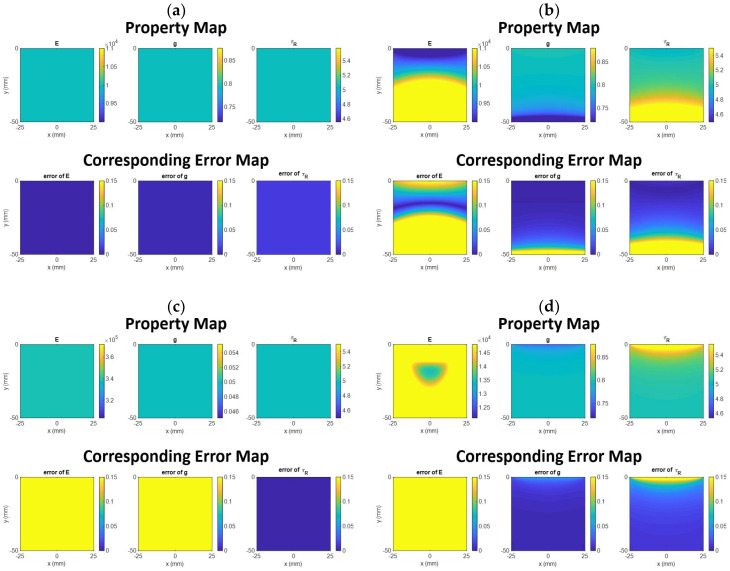

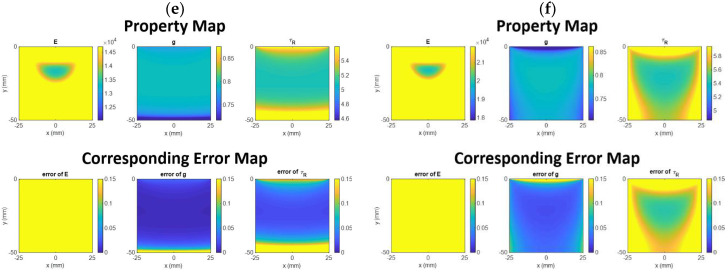
The 2D spatial distribution and corresponding error maps. In the error map, the yellow color means the error is larger than 10%. (**a**) Simulation test 1. (**b**) Simulation test 2. (**c**) Simulation test 3. (**d**) Simulation test 4. (**e**) Simulation test 5. (**f**) Simulation test 6.

Figure 6 shows the 2D spatial distribution map and corresponding error map for each mechanical property in each simulation test. Table 1 shows the percentage of the region in the 2D spatial distribution map consisting of elements with a simulation value within ±10% of the theoretical value for each simulation test.

In the first simulation test (when the uniform compressional pressure is exerted on the entire top surface, and the bottom is fixed along the vertical direction while the side is not fixed), the 2D distribution map for each mechanical property is perfectly homogeneous, as is the corresponding error map. The error value at each element in the error map is nearly zero. This means that all three mechanical properties can be accurately measured in this case.

In the second simulation test (when the uniform compressional pressure is exerted on the entire top surface, and the bottom is fixed along all directions while the side is not fixed), g can be accurately measured in most of the region (92.42%), except for a small region close to the bottom. $\tau_{R}$ can be accurately measured in 77.5% of the entire region, except for a region extending from the bottom to near the depth of 40 mm. For E, it can only be accurately measured in 37.28% of the entire region, between the depth of 5 to 25 mm approximately, and there is a significant region where each element in this region has an error larger than 10%.

In the third simulation test (when the uniform compressional pressure is exerted on the entire top surface, and the bottom is fixed along the vertical direction while the side is fixed along the horizontal direction), the 2D distribution map for each mechanical property is perfectly homogeneous. However, only $\tau_{R}$ can be accurately measured, while E and g cannot be since each element in the entire region has an error larger than 10%.

In the fourth and fifth simulation tests (when the uniform compressional pressure is exerted on the half of the top surface, and the bottom is fixed along the vertical direction), g and $\tau_{R}$ can be accurately measured, except for a small region close to the top and/or bottom. E cannot be accurately measured since each element in the entire region has an error larger than 10%.

In the sixth simulation test (when the uniform compressional pressure is exerted on the half of the top surface, and the bottom is fixed along the vertical direction while the side is fixed along the horizontal direction), g can be accurately measured in most of the region (96.18%). For $\tau_{R}$, it can only be accurately measured in 23.24% of the region. E cannot be accurately measured since each element in the entire region has an error larger than 10%.

## 4. Discussion

The findings of the present finite element analysis study provide a reference for experimental phantom designs regarding loading and boundary conditions, as well as guidance towards validating the experimental results of a compressional viscoelastography system, such as the one in the literature [33,34]. The most important finding is that the accuracy of compressional viscoelastography for measuring the viscoelastic properties of materials is only excellent if the compressional pressure is exerted on the entire top surface of the sample, as well as if the bottom of the sample is fixed just along the vertical direction. These findings imply that in an experimental validation study, the phantom design should take into account that the surface area of the pressure plate must be equal to or larger than that of the top surface of the sample, and the sample should be placed directly on the testing platform without any fixation (such as a sample container). However, in an experimental design, the sample may slip horizontally under loading if there is no fixation to stabilize the sample. Fortunately, the slip can be prevented by using a pressure plate with a surface area significantly larger than that of the top surface of the sample.

**Figure 7:**
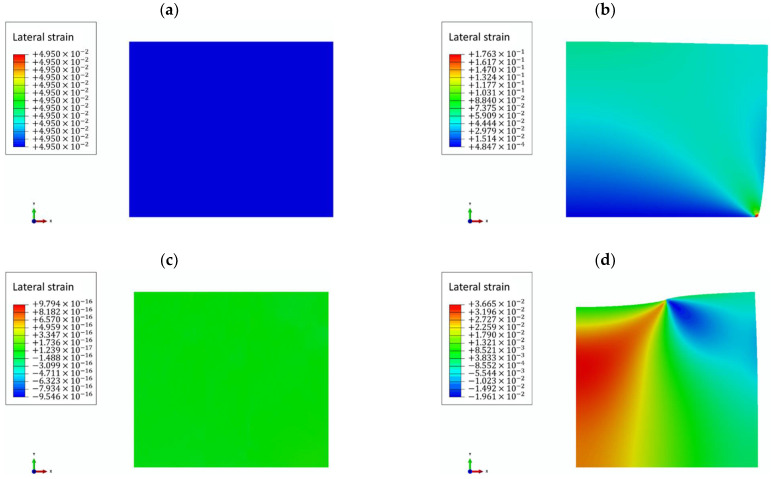

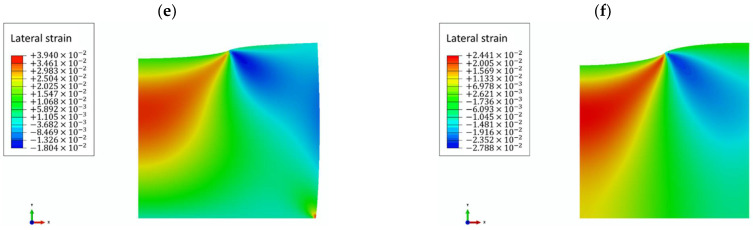
The distribution of horizontal strains in each simulation test. (**a**) Simulation test 1. (**b**) Simulation test 2. (**c**) Simulation test 3. (**d**) Simulation test 4. (**e**) Simulation test 5. (**f**) Simulation test 6.

In the present study, it was found that the loading and boundary conditions in computational simulations of compressional viscoelastography severely affect the accuracy of measuring the modulus of elasticity, E. If the compressional pressure is exerted on half of the top surface of the sample and the boundary condition is more complex than the abovementioned optimal condition (the bottom of the sample is fixed just along the vertical direction), E is difficult measure accurately. This implies that if the area of the pressure plate is smaller than that of the top surface of the sample, and if the sample is secured by any external fixation method (such as the sample container shown in Figure 2b), E may not be accurately measured. In these kinds of conditions, there will be horizontal strains occurring within the sample. The theory used in the present study for analyzing the creep curve, Equation (1), is a one-dimensional model that only considers the strain component along the vertical direction. Therefore, if there are horizontal strains within the sample, Equation (1) may not yield precise measurements; the larger the horizontal strains, the greater the errors. This may be the reason why an accurate measurement for E cannot be achieved in the conditions where horizontal strains can develop within the sample. In addition, the distribution of horizontal strains affects the homogeneity of the 2D spatial distribution map of E. Figure 7 shows the distribution of horizontal strains in each simulation test. It can be observed that every test has horizontal strains. If the distribution of horizontal strains is homogeneous, such as that in the first and third tests, the 2D spatial distribution map of E is homogeneous. On the other hand, if the distribution of horizontal strains is nonhomogeneous, the 2D spatial distribution map of E is nonhomogeneous. Therefore, for an accurate measurement of E in more complex loading and boundary conditions, strain components along both horizontal and vertical directions must be considered simultaneously, and a multi-dimensional model must be used for data analysis. The measurements of $\tau_{R}$ and g are also significantly affected by loading and boundary conditions. It is interesting to note that when the compressional pressure is exerted on half of the top surface of the model, the measurement of g is less affected by the boundary condition. This means that g can always be accurately measured no matter what the boundary condition is. On the other hand, the more complex the boundary condition, the more difficult the measurement of $\tau_{R}$.

The initial intention for developing compressional viscoelastography was to measure the viscoelastic properties of breast tissues in vivo for the diagnosis of breast tumors [33,34]. Unfortunately, in the present study, it has been found that the loading and boundary conditions in computational simulations of compressional viscoelastography severely affect the measurement accuracy. The measurement can only be accurate if the compressional pressure is exerted on the entire top surface of the sample, as well as if the bottom of the sample is fixed just along the vertical direction. However, the loading and boundary conditions could be much more complex than these optimal conditions when measuring real tissues in vivo. Therefore, the findings indicate that when applying compressional viscoelastography to real tissues in vivo, consideration should be given to the representative loading and boundary conditions. Further studies are needed to investigate the measurement accuracy of compressional viscoelastography on both biomaterials and real tissues in vivo.

Finite element analysis has been applied to investigate the performance or the effects of system parameters of ultrasound elastography [37,38,39,40]. There are also some valuable studies using finite element analysis to explore magnetic resonance elastography [41,42,43,44]. However, to the best of our knowledge, there are only two studies that have used finite element analysis to investigate the performance of ultrasound compressional viscoelastography on the measurement of the viscoelastic properties of materials [34,45]. It is the authors’ belief that more studies are needed to investigate the performance of ultrasound compressional viscoelastography to justify its usefulness in clinical or biomedical applications.

Since the present study is based on finite element analysis, it is very important to further discuss and explain the simulation settings to ensure that the simulation results are understood and applied properly. First, the finite element model used in this study was a simple axisymmetric model. The findings could be successfully applied on biomaterials since they are often designed as an axisymmetric cylinder. In the future, a more complex shape for the model should be considered so that the findings can be more accurately applied to samples with different geometries. Second, the magnitude of applied force could be different across various simulation tests since the magnitude of compressional pressure was the same, but the area over which it was applied might vary across test. The reason for applying a constant pressure (but not a constant force) on each test is that the pressure can be regarded as the force normalized by the area over which it is applied. Therefore, by applying a constant pressure on each test, similar orders of magnitude of stress and strain can be induced within the model. Third, the volume of the model relative to the compression area is an important parameter, but this parameter was outside the scope of this study. During a compression test by compressional viscoelastography, if the aspect ratio (diameter/thickness) of the model is much smaller than one, buckling could occur. Therefore, in order to prevent buckling, the aspect ratio of the model must be larger than one. This is why the axisymmetric model was designed to have a constant volume with a radius of 50 mm and a thickness of 50 mm (the associated aspect ratio is two).

The present study has some further limitations: (1) not all variables relevant to compressional viscoelastography were investigated. In addition, no noise or imaging uncertainties were considered in the finite element analysis in this study; in reality, the signal of noise and vibration may affect the accuracy of the system. Therefore, the findings of this study only can provide suggestions and references for experimental phantom designs regarding loading and boundary conditions, but cannot provide global guidance for every technical aspect relevant to compressional viscoelastography. (2) Only two types of loading conditions were investigated. It will be valuable to conduct a more detailed parametric analysis to quantitatively investigate the relationship between the loading condition and the area of the image region with accurate measurements. (3) Only homogeneous materials were investigated, and an inclusion phantom was not considered. (4) There was no experiment to validate the finite element analysis results of this study. Both simulations and experiments are important and have their own merits and limitations. Simulation offers the possibility and convenience to exactly control a condition (for example, to set the viscoelastic properties of materials as specific values) for the analysis of a great variety of conditions, and can show a relative and general trend to provide guidance for experimental design. Experiments provide real data showing what happens in reality. In the future, it is important to investigate if the same results can be observed in real experiments.

## 5. Conclusions

In conclusion, the findings of the present simulation study will provide a reference for experimental phantom designs regarding loading and boundary conditions, as well as guidance towards validating the experimental results of compressional viscoelastography (viscoelastic creep imaging using external compression as the source of excitation). The results show that loading and boundary conditions in computational simulations of compressional viscoelastography can severely affect the measurement accuracy of the viscoelastic properties of materials. The measurement can only be accurate if the compressional pressure is exerted on the entire top surface of the sample, and if the bottom of the sample is fixed just along the vertical direction. These findings imply that, in an experimental validation study, the phantom design should take into account that the surface area of the pressure plate must be equal to or larger than that of the top surface of the sample, and the sample should be placed directly on the testing platform without any fixation (such as a sample container). These findings indicate that when applying compressional viscoelastography to real tissues in vivo, consideration should be given to the representative loading and boundary conditions.

## Figures and Tables

**Table 1:** The percentage of the region in the map consisting of elements with the simulation value within ±10% of the theoretical value set in ABAQUS. The larger this percentage, the more accurate the measurement.

Simulation Test Number	Percentage of the Region in the Map Having Accurate Measurement (%)		
	*E*	$\tau_{R}$	g
1	100	100	100
2	37.28	77.50	92.42
3	0	100	0
4	0	93.84	100
5	0	83.16	95.94
6	0	23.24	96.18

## Data Availability

The datasets relevant to this study are available on request to the corresponding author.

## Appendix A

Simulation test 1 was also used to investigate the validity of compressional viscoelastography. In this investigation, we studied three groups of materials with different mechanical properties (Table A1). In group 1, E = 10, 20, and 30 kPa, $\tau_{R}$ = 5 s, and g = 0.8. In group 2, $\tau_{R}$ = 1, 5, and 10 s, E = 10 kPa, and g = 0.8. In group 3, g = 0.4, 0.6, and 0.8, E = 10 kPa, and $\tau_{R}$ = 5 s. For the map of each mechanical property, the simulation value (the median of the values of all elements) is compared to the theoretical value (the value of that mechanical property set in ABAQUS) using the following equation:

$$\mathrm{error}=\frac{\left|\mathrm{simulation}\;\mathrm{value}-\mathrm{theoretical}\;\mathrm{value}\right|}{\mathrm{theoretical}\;\mathrm{value}}$$

Table A1 shows the results. It can be observed that the theoretical and simulation values are very close for each case. Figure 6a shows the 2D distribution map of each mechanical property and the corresponding error map for simulation test 1. It can be observed that the map for each mechanical property is perfectly homogeneous, as is the corresponding error map. The error for each element in the error map is nearly zero. The results show that the measurement is accurate, justifying the validity of compressional viscoelastography.

**Table A1:** The results of investigating the validity of compressional viscoelastography. It can be observed that the theoretical and simulation values are very close for each case.

	Theoretical Values			Simulation Values			Error (%)		
Material Number	*E*	$\tau_{R}$	g	*E*	$\tau_{R}$	g	*E*	$\tau_{R}$	g
Group 1									
1	10,000	5	0.8	10,001	5.067	0.797	0.01	1.35	0.34
2	20,000	5	0.8	20,003	5.068	0.797	0.01	1.37	0.34
3	30,000	5	0.8	30,006	5.069	0.797	0.02	1.38	0.34
Group 2									
1	10,000	1	0.8	10,001	1.014	0.797	0.01	1.35	0.34
2	10,000	5	0.8	10,001	5.067	0.797	0.01	1.35	0.34
3	10,000	10	0.8	10,001	10.135	0.797	0.01	1.34	0.34
Group 3									
1	10,000	5	0.4	10,000	5.011	0.399	0.00	0.23	0.34
2	10,000	5	0.6	10,000	5.025	0.598	0.00	0.51	0.34
3	10,000	5	0.8	10,001	5.067	0.797	0.01	1.35	0.34
